# Frailty modifies the effect of polypharmacy and multimorbidity on the risk of death among nursing home residents: Results from the SHELTER study

**DOI:** 10.3389/fmed.2023.1091246

**Published:** 2023-02-01

**Authors:** Maria Beatrice Zazzara, Emanuele Rocco Villani, Katie Palmer, Daniela Fialova, Andrea Corsonello, Luca Soraci, Domenico Fusco, Maria Camilla Cipriani, Michael Denkinger, Graziano Onder, Rosa Liperoti

**Affiliations:** ^1^Fondazione Policlinico Universitario A. Gemelli IRCCS (Istituto di Ricovero e Cura a Carattere Scientifico), Polo Interdipartimentale Scienze Dell'Invecchiamento, Neuroscienze, Testa-collo ed Ortopedia, Rome, Italy; ^2^Università Cattolica del Sacro Cuore, Polo Interdipartimentale Scienze Dell'Invecchiamento, Neuroscienze, Testa-collo ed Ortopedia, Rome, Italy; ^3^Department of Geriatrics and Gerontology, 1st Faculty of Medicine, Charles University, Prague, Czechia; ^4^Unit of Geriatric Medicine, IRCCS INRCA (Istituto Nazionale Ricovero e Cura Anziani), Dipartimento di Medicina Interna e Terapia Medica, Cosenza, Italy; ^5^AGAPLESION Bethesda Ulm, Geriatric Research Ulm University and Geriatric Center Ulm/Alb Donau, Ulm, Germany

**Keywords:** drugs, hyperpolypharmacy, frailty, long-term care facility, mortality

## Abstract

**Background:**

Frailty, disability, and polypharmacy are prevalent in nursing home (NH) residents, often co-occurring with multimorbidity. There may be a complex interplay among them in terms of outcomes such as mortality. Aims of the study were to (i) assess whether nursing home residents with polypharmacy (5–9 medications) or hyperpolypharmacy (≥10 drugs), have an increased risk of death and (ii) whether any association is modified by the co-presence of frailty or disability.

**Methods:**

Cohort study with longitudinal mortality data including 4,023 residents from 50 European and 7 Israeli NH facilities (mean age = 83.6 years, 73.2% female) in The Services and Health for Elderly in Long Term care (SHELTER) cohort study. Participants were evaluated with the interRAI-LongTerm Care assessment tool. Frailty was evaluated with the FRAIL-NH scale. Hazard ratio (HR) of death over 12 months was assessed with stratified Cox proportional hazards models adjusted for demographics, facilities, and cognitive status.

**Results:**

1,042 (25.9%) participants were not on polypharmacy, 49.8% (*n* = 2,002) were on polypharmacy, and 24.3% (*n* = 979) on hyperpolypharmacy. Frailty and disability mostly increased risk of death in the study population (frailty: HR = 1.85, 95%CI 1.49–2.28; disability: HR = 2.10, 95%CI 1.86–2.47). Among non-frail participants, multimorbidity (HR = 1.34, 95%CI = 1.01–1.82) and hyperpolypharmacy (HR = 1.61, 95%CI = 1.09–2.40) were associated with higher risk of death. Among frail participants, no other factors were associated with mortality. Polypharmacy and multimorbidity were not associated with mortality after stratification for disability.

**Conclusions:**

Frailty and disability are the strongest predictors of death in NH residents. Multimorbidity and hyperpolypharmacy increase mortality only in people without frailty. These findings may be relevant to identify patients who could benefit from tailored deprescription.

## Introduction

Frailty is broadly defined a “clinical state characterized by a decrease of an individual's homeostatic reserves and is responsible for enhanced vulnerability to endogenous and/or exogenous stressors” (1). Multiple operational definitions of frailty are available in literature (*x*). The most frequently used definition (2) focuses on the evaluation of five domains (nutritional status, energy, physical activity, mobility, and strength) to identify the frail phenotype. Independently of the adopted definition, frailty has been repeatedly associated with several negative health outcomes such as fracture risk (3), hospitalization (4), disability (5), multimorbidity (6), and medication harm (7). A recent meta-analysis of studies using the Frailty Index, reported that frailty is a significant predictor of mortality (8). It has been also suggested that polypharmacy may be an explanatory factor of the association between frailty and mortality in older individuals (9). Polypharmacy is common in people with frailty (10). Both frailty and prefrailty significantly predict nursing home (NH) placement (11) and a meta-analysis reported a very high prevalence of both conditions (40.2 and 52.3%, respectively) in NH patients (12), especially among women (13). More than 90% of patients awaiting NH placement have at least one potentially inappropriate medication (PIM) and 65% are eligible for the application of STOPPFrail criteria (14) (Screening Tool of Older Persons Prescriptions in Frail adults with limited life expectancy), which is designed to help physicians in deprescribing medications in older patients with frailty and limited life expectancy. The interplay between polypharmacy, multimorbidity, and frailty is potentially complex (15). For example, number of drugs can be higher than number of comorbidities, they both can be determinants of frailty, and disease-drug and drug-drug interactions are frequent. Given the high prevalence of these conditions in NH patients, it is of interest to see how these common phenomena affect mortality in this setting of care.

The aims of the current paper are to (i) assess whether nursing home residents with polypharmacy (5–9 medications) or hyperpolypharmacy (≥10 drugs), have an increased risk of death and (ii) whether any association is modified according to the presence of frailty or disability and multimorbidity.

## Methods

### Study design

This longitudinal cohort study is a secondary analysis based on data from the Services and Health for Elderly in Long Term care (SHELTER) study (16). The SHELTER study is a longitudinal cohort study that was conducted between 2009 and 2011 and it includes information on 4,156 participants from 50 European nursing home facilities (10 in Czech Republic, 9 in England, 4 in Finland, 4 in France, 9 in Germany, 10 in Italy, and 4 in the Netherlands) and from 7 facilities in Israel.

### Study participants

Participants were randomly selected among older adults residing in participating NHs at the beginning of the study and those admitted in the 3 month enrolment period following the initiation of the study, based on their willingness to participate in the SHELTER study.

The original aim of the SHELTER study was to validate the use of the interRAI-LongTerm Care assessment tool (InterRAI-LTCF) as a methodology to assess provision of care in NH in Europe All participants were evaluated by trained assessors with the InterRAI-LTCF, which includes more than 350 elements, covering sociodemographics, clinical items about physical and cognitive status, and clinical diagnoses. The tool also collects information about clinical signs, symptoms, diseases, and treatments (16).

### Statement of ethics

Ethical approval for the study was obtained in all countries according to country-specific agreements, on the behalf of the ethical committee of the Catholic Unviersity of the Sacred Heart, approval number P/220/CE/2009. Residents were invited to take part in the study and were free to decline participation. Written informed consent was obtained from participants, or their legal guardian, to participate in the study.

### Frailty assessment

Frailty was evaluated according to the FRAIL-NH scale, a tool specifically developed for nursing homes (17). The FRAIL-NH is easy to administer and based on seven potentially reversible conditions of frailty: *F* = fatigue, *R* = resistance, *A* = ambulation, *I* = incontinence, *L* = weight loss, *N* = nutritional approach, *H* = help with dressing. The overall score ranges from 0 to 14. Frailty was defined as score equal or >8 at the FRAIL-NH scale. In the paper that describes thoroughly the process of codfication, FRAIL-NH codification and application has been demonstrated to be a reliable indicator of frailty compared to a comprehensive geriatric assessment for nursing home residents in the SHELTER population (18).

### Disability assessment

To evaluate the presence of disability, participants' functional status has been recorded through the Activities of Daily Living (ADL) Hierarchy scale (19). The ADL Hierarchy scale groups ADL according to the stage of the disablement process in which they occur, assigning lower scores to early-loss ADLs (i.e dressing, personal hygiene, and toilet use) than to late-loss ADLs (i.e., transfer, locomotion, bed mobility, and eating). The ADL Hierarchy Scale ranges from 0 (independent) to 6 (total dependence). Disability was deemed present in the presence of ADL Hierarchy Scale score 5–6, and absent in presence of ADL Hierarchy scale score 0–4.

### Medications and polypharmacy

Information on medications was collected at baseline assessment using the dedicated InterRAI-LTCF section according to their Anatomical Therapeutic and Chemical code. Polypharmacy was defined as concurrent use of 5–9 medications and hyperpolypharmacy was defined as the concurrent use of 10 or more drugs (20).

### Covariates

Covariates were assessed through specific items from the InterRAI-LTCF. Cognitive status was assessed with the Cognitive Performance Scale (CPS) (21). This scale assesses memory impairment, level of consciousness, and executive function, with scores ranging from 0 (intact) to 6 (very severe impairment). Cognitive impairment was categorized as follows: none-borderline intact (CPS score 0–1), mild to moderate (CPS score 2–4), and severe (CPS score 5–6). The Depression Rating Scale (DRS) was used to assess the presence of depressive symptoms, and a cut-off score ≥3 was used to indicate the presence of a clinically significant depression (22). Information on the presence of following chronic conditions was also collected: heart failure, ischemic heart disease, Parkinson's disease, stroke, diabetes, COPD, cancer, and dementia. Multimorbidity was defined as the concurrent presence of two or more different comorbidities (23), including also dementia and depression.

### Mortality

Participants were followed over 1 year during their residence in the nursing homes and all deaths were recorded. No information regarding causes of death was gathered. Time to death was considered as the date of the first assessment until the date of death. An extra record was made in case of discharge from the facility and cases were censored at this date. In survival analyses, death for any cause was considered as the outcome and discharge from the facility as censoring events. For remaining participants, time was censored at 12-months.

### Statistical analysis

Baseline characteristics (mean and standard deviation or number and percentage) of participants were compared between frail and non-frail participants using analyses of variance and Tukey *post-hoc* for normally distributed variables, while chi-square and *post-hoc Z*-test were used for dichotomous variables. A two-tail *p*-value < 0.05 was considered significant.

Collinearity was tested according to Pearson correlation coefficient and Variance Inflation Factor (VIF). A VIF > 10 indicates severe collinearity. For FRAIL-NH and ADL Hierarchy scale, that share 3 items (ambulation, help with dressing and incontinence), VIF was 4.1 and Pearson's *R*^2^ was 0.678, suggesting collinearity between the two scales. On the contrary, no collinearity was detected for multimorbidity and polypharmacy (VIF = 2.1, *R*^2^ = 0.033). Hence, two different models to assess differences in mortality according to polypharmacy status were performed: the first one exploring frail and non-frail patients, the second one exploring patients with and without disability in the ADLs. Survival curves were plot through Kaplan–Meier methodology and the Log-Rank test was used to assess difference among survival curves. Shared frailty Cox proportional hazard regression models were used to evaluate the effect of polypharmacy on time to death in both frail vs. non-frail patients and those with and without disability, while accounting for potential random effect due to data clustering within NHs (24). Potential confouders included age, gender, and variables that were associated with polypharmacy at the univariate analysis. To exclude departure from proportionality assumption, the log-log survival function was examined. All analyses were performed by using STATA version 14.0 for Windows.

## Results

At baseline, from the initial sample of 4,156 participants, 133 (3.2%) were excluded due to missing data, leading to a final sample of 4,023 participants. The sociodemographic and clinical characteristics of participants have been described for the overall study population and according to the number of medications used (Table 1). The mean age in the overall study population was 83.6 years and almost two-thirds were female. Almost half (47%) of the population were classified as frail and 40.6% had multimorbidity. A quarter (*n* = 1,042, 25.9%) of participants were not on polypharmacy regimen, while a half (*n* = 2,002, 49.8%) were on polypharmacy and a quarter (*n* = 979, 24.3%) were on hyperpolypharmacy. Laxatives, antiulcer medications, psychotropic drugs (such as benzodiazepines, antidepressants, and antipsychotics), and diuretics were the most common classes prescribed in the study population.

**Table 1:** Baseline characteristics of the study population.

**Variable**	**All (*n* = 4,023)**	**0–4 drugs (*n* = 1,042)**	**5–9 drugs (*n* = 2,002)**	**10+ drugs (*n* = 979)**	***p*-value^A^**
	***n*** **(%)**	***n*** **(%)**	***n*** **(%)**	***n*** **(%)**	
Age (mean, SD)	83.6 (9.4)	83.4 (10.3)	83.7 (9.1)	83.3 (9.4)	0.461
Female	2,945 (73.2)	765 (73.4)	1,478 (73.8)	702 (71.7)	0.463
Parkinson's disease	276 (6.9)	55 (5.3)	132 (6.6)	89 (9.1)^b^	0.003
Stroke	886 (22.1)	195 (18.9)	456 (22.8)^b^	234 (24.0)^b^	0.012
Heart failure	708 (17.7)	98 (9.5)	360 (18.0)^b^	250 (25.8)^b^	<0.001
Cancer	435 (10.9)	74 (7.1)	230 (11.5)^b^	131 (13.4)^b^	<0.001
Dementia	1,445 (36.1)	403 (38.9)	763 (38.2)	279 (28.7)^b^	<0.001
Depressive symptoms	956 (23.9)	157 (15.2)^a^	495 (24.8)^b^	304 (31.2)^c^	<0.001
Multimorbidity	1,636 (40.6)	287 (27.9)^a^	853 (42.8)^b^	496 (52.9)^c^	<0.001
Frailty	1,878 (47.0)	533 (51.6)	919 (46.2)^*b*^	426 (44.1)^b^	0.002
Cognitive impairment (CPS)					<0.001
0–1: intact/borderline	1,880 (46.7)	405 (40.2)^a^	921 (46.4)^b^	554 (57.0)^c^	
2–4: mild-moderate	851 (21.2)	195 (19.4)	434 (21.9)	222 (22.8)	
5–6: severe	1,234 (30.7)	407 (40.4)^a^	631 (31.8)^b^	196 (20.2)^c^	
Disability (ADLs)					0.147
0–4: absent	2,778 (69.2)	743 (71.7)	1,368 (68.5)	667 (68.2)	
5–6: present	1,234 (30.8)	294 (28.4)	629 (34.5)	311 (31.8)	

ADL, activities of daily living; CPS, cognitive performance scale.

Each subscript letter indicates polypharmacy categories whose proportions are comparable at level 0.05.

A Age assessed with Anova with Tukey *post-Hoc*, all other variables assessed with Pearson's Chi-square with Z-test (Bonferroni *post-Hoc*).

Comorbidities such as Parkinson's disease, stroke, heart failure, and cancer were more common among patients on polypharmacy or hyperpolypharmacy compared to those who were not. Multimorbidity was more common in participant on polypharmacy (42.8%) or hyperpolypharmacy (52.9%) than people taking 0–4 drugs (27.9%) (*p* < 0.001). Dementia and poor cognitive status were inversely associated with being on polypharmacy or hyperpolypharmacy (*p* < 0.001). The proportion of residents with disability (ADL score 5–6) was comparable among polypharmacy groups (*p* = 0.147). On the contrary, frailty was less common in participants on polypharmacy (46.2%) or hyperpolypharmacy (44.1%) compared to the group of those taking <5 drugs (51.6%) (*p* = 0.002).

Overall, 761 (18.9%) participants died during follow-up and 262 (6.5%) moved out of the nursing home and were censored. Overall the incidence rate of death in the study population was 0.21 persons/year. Table 2 shows factors associated with death during 1-year follow-up. After adjusting for sociodemographics and potential confounders (facilities, cognitive status), frailty and disability were associated with the highest risk of death (frailty: HR = 1.85, 95%CI 1.49–2.28; disability: HR = 2.10, 95%CI 1.68–2.47), while both polypharmacy and hyperpolypharmacy and cognitive status showed no effect on mortality.

**Table 2:** Factors associated with mortality during 1-year follow-up (*n* = 1,187).

**Variables**	**Crude HR (95% CI)**	Adjusted HR (95% CI)^*^
Age	1.03 (1.02–1.04)	1.04 (1.03–1.05)
Female sex	0.76 (0.64–0.89)	0.69 (0.59–0.83)
Frailty	2.37 (1.99–2.80)	1.85 (1.49–2.28)
**Number of drugs**		
0–4: no polypharmacy	1	
5–9: polypharmacy	1.08 (0.87–1.34)	1.09 (0.90–1.33)
≥10: hyperpolypharmacy	1.20 (0.88–1.64)	1.29 (0.98–1.87)
Multimorbidity	1.30 (1.13–1.50)	1.12 (0.93–1.35)
**Cognition (CPS)**		
0–1: intact/borderline	1	1
2–4: mild-moderate	1.46 (0.93–1.76)	1.16 (0.93–1.44)
5–6: severe	1.87 (1.06–2.02)	1.52 (0.96–1.88)
**Disability**		
ADL 0–4: absent	1	1
ADL 5–6: present	2.35 (1.95–2.84)	2.10 (1.68–2.47)

ADL, activities of daily living; CPS, cognitive performance scale.

* Adjusted for demographics, facilities are the shared frailty.

**Figure 1:**
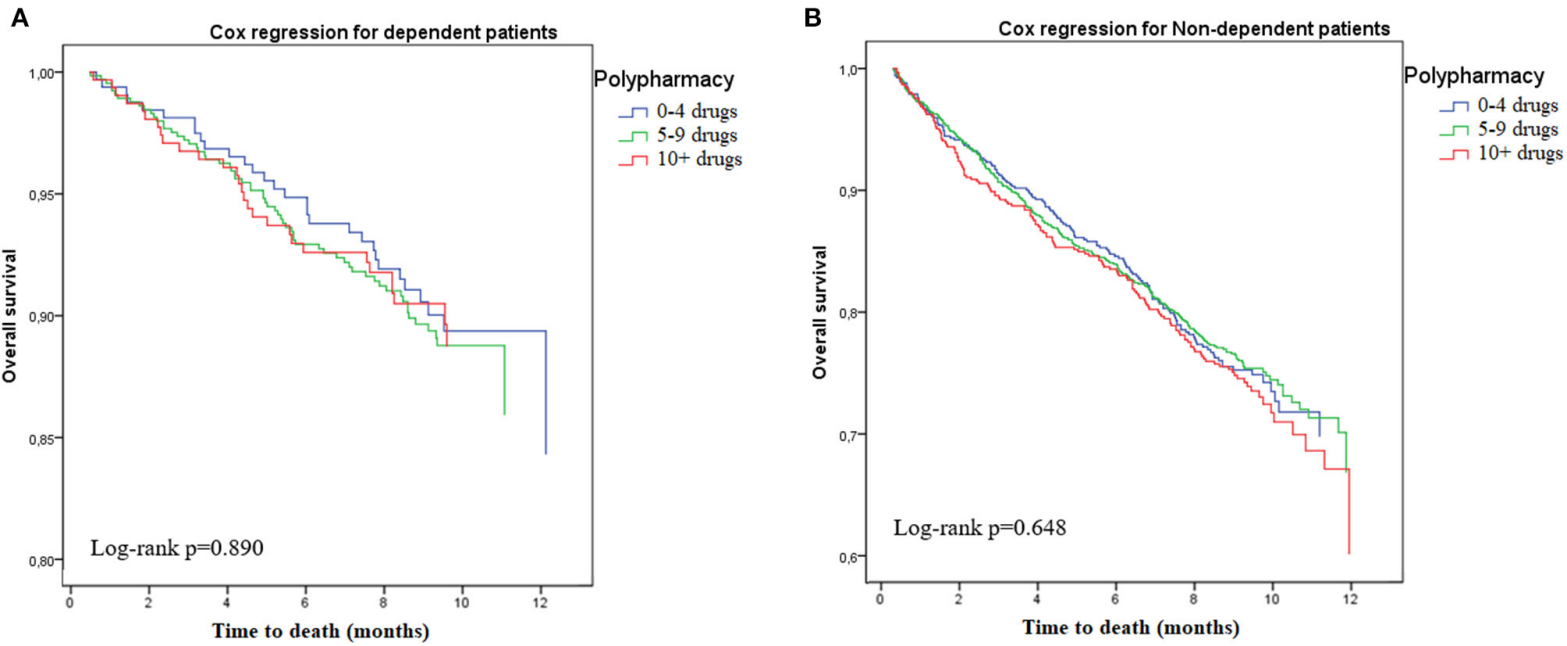
Risk of death according to polypharmacy status, stratified by disability. **(A)** Dependent. **(B)** Non-dependent.

**Figure 2:**
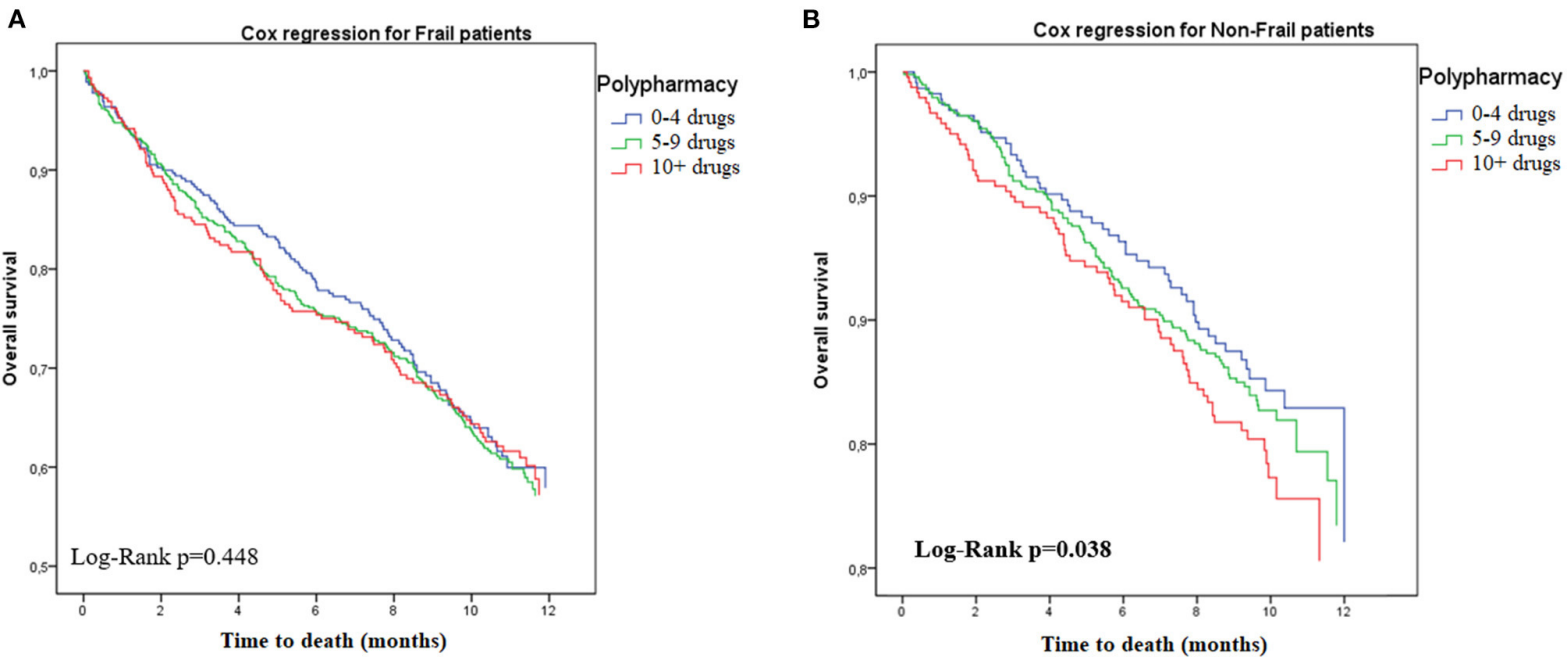
Risk of death according to polypharmacy status, stratified by frailty. **(A)** Frail. **(B)** Non-frail.

Table 3 shows the results of the Cox proportional hazards regression after stratification for frailty status (see also Figures 1, 2 for the Kaplan-Meyer survival curves for polypharmacy). Among non-frail participants, multimorbidity (adjusted HR 1.34, 95%CI = 1.01–1.82) and hyperpolypharmacy (adjusted HR 1.61, 95%CI = 1.09–2.40) were associated with a higher risk of death during the follow-up. Among frail participants, no other factors were significantly associated with death. Conversely, both polypharmacy and multimorbidity were not associated with a different risk of death after stratification for disability.

**Table 3:** Predictors of 1-year mortality stratified by frailty and disability status.

**Variables**	**Frail**		**Non-frail**		**Disabled**		**Non-disabled**	
	**Adj. HR**	**(95% CI)**	**Adj. HR**	**(95% CI)**	**Adj. HR**	**(95% CI)**	**Adj. HR**	**(95% CI)**
Multimorbidity	1.02	(0.81–1.28)	1.34	(1.01–1.82)	1.37	(0.90–2.09)	1.10	(0.90–1.35)
**Number of drugs**								
0–4: no polypharmacy	1		1		1		1	
5–9: polypharmacy	1.14	(0.91–1.43)	1.08	(0.77–1.52)	1.02	(0.64–1.64)	1.09	(0.89–1.34)
≥10: hyperpolypharmacy	1.29	(0.96–1.89)	1.61	(1.09–2.40)	1.18	(0.86–1.80)	1.31	(0.98–1.74)

Adjusted for demographics, Cognitive Performance Scale, facilities are the shared frailty.

## Discussion

In this longitudinal study of older nursing home residents, we found that hyperpolypharmacy and multimorbidity both increase the risk of death, but only in people without frailty, even after adjustment for demographics, facilities, comorbidities, and cognitive status. Person without frailty who had multimorbidity had 35% increased risk of death compared to those without multimorbidity. There was a 29% increased mortality risk in people with hyperpolypharmacy compared to those without polypharmacy, within the non-frail stratum.

Chart review of patients in the last year of life suggest that there is a continued prescribing of futile medications (25). However, our findings are in contrast to the SHARE study, which reported that people with both frailty and polypharmacy have an increased risk of death over a 30-month period (26), similar to a British study (27). Further, a Spanish study found opposite findings to ours; polypharmacy was associated with mortality in frail and prefrail older adults, but not in non-frail individuals (28). It should be noted that our population included only nursing home residents not the general population and this may partly explain our findings. Nursing home residents already have a high vulnerability and complex care needs and may have specific characteristics that affect the interplay between frailty and polypharmacy. A previous paper from the SHELTER study found that frailty is associated with less polypharmacy and with higher prevalence of symptomatic drugs use among residents (18). Further, drug patterns are different among frail and non-frail persons, with the latter showing higher prevalence of disease-modifying drugs, while frail patients show lower prevalence of drugs related to adverse health-outcomes (18).

Frailty is associated with short-term mortality also in older people without multimorbidity (29) and disability, rather than multimorbidity, has been shown to be predictive of death in older adults (30, 31). It is possible that, in our sample of complex older NH residents, frailty is one of the main determinant of negative outcomes including death while, in participants without frailty, the comorbidities and the related treatment burden have an impact on prognosis. The effect of these conditions could the be flattened by the presence of frailty and the association between polypharmacy and mortality only becomes evident only in absence of frailty.

Future research is needed to replicate our findings and identify mechanisms behind the results (25–28). Indeed, it is important to assess the specific vulnerability and characteristics of nursing home residents to verify why there is a different mortality risk associated with frailty and polypharmacy in this population. It may also be relevant to assess how the interplay between these variables differs in younger individuals; frailty is a predictor of mortality also in younger adults (aged 37–73) (32) and is associated with multimorbidity even at these ages. Further, it may be of interest to assess whether interventions such a deprescribing or geriatric cognitive assessment can modify frailty or polypharmacy and alter subsequent risk of death, both in frail and non-frail individuals. Within frail individuals, a systematic review concluded that deprescribing could be safe, feasible, well tolerated and can lead to important benefits (33). However, evidence is still conflicting on whether desprescribing will have any effect on mortality as well as several factors are involved into the deprescribing process (34). A STOPPFrail-guided deprescribing plan in older nursing home residents with frailty and polypharmacy was successful in reducing polypharmacy but did not affect mortality, although the study may not have been sufficiently powered to assess this (35). As our study found higher mortality associated with hyperpolypharmacy in non-frail individuals, the question is whether deprescribing should be prioritized in individual's without frailty. One study comparing nursing home and home-based settings reported that nursing homes provide a highly suitable scenario to carry out a periodic medication review as it is more feasible to apply the review recommendations (36). Polypharmacy is also related to other relevant outcomes in older adults including quality of life. Appropriate deprescribing interventions should be then promoted in the older population, regardless of the frailty status (37).

On the other hand, NHs resident could ondergo other interventions in order to ameliorate their health outcomes, besides deprescribing. That is why it is worth it to thoroughfully assess nursing home patients both with and without frailty: one randomized control study found that an outpatient intervention with Comprehensive Geriatric Assessment (CGA) may both delay the progression of frailty and may contribute to the improvement of frail patients in older persons with multimorbidity (38). A CGA should also assess emotional wellbeing that is strongly associated with frailty (39). Moreover, multidimensional interventions in geriatric settings are likely to be effective in the care of hospitalized frail elderly (40).

### Strengths and limitations

In the current study we defined frailty with the FRAIL-NH, although there are a large range of definitions and tools in the literature (41). FRAIL-NH has demonstrated good agreement with other well-established but more complex frailty scales and has a value for guiding care for frail residents in nursing homes (40). A major limitation is that frailty status was dichotomized, so prefrail status was not evaluated, which may have been of interest. Another limitation is that drug use was measured with InterRAI-LCTF, which is not a tool specifically focused on medications. We recorded only medications used within the previous 3 days, which may affect the accuracy of the polypharmacy and hyperpolypharmacy variables. Moreover, other health-related outcomes that could benefit from pharmacological assessment (i.e., deprescribing) were not evaluated in the study. Further, the data come from 2009 to 2011 and the sample was not meant to be nationally representative, so care should be taken before generalizing the results to nursing home residents today in different countries. A strength of our study was the follow-up collection of mortality data but we were, unfortunately, not able to assess cause of death. The InterRAI-LCTF data collection at baseline also only looked at one point in time and it is possible that frailty, multimorbidity, and polypharmacy status changed over the follow-up time.

## Conclusions and implications

In conclusion, among nursing home residents in the SHELTER study multimorbidity and polypharmacy increased the risk of death only in people without frailty. This may be relevant for planning which vulnerable older people should be targeting for deprescription. However, more research is needed to verify whether these findings are replicated in other health care settings and to identify the mechanisms behind the difference in the risk of negative health outcomes and death according to frailty status.

## Data availability statement

All data generated or analyzed during this study are included in this article. Further enquiries can be directed to the corresponding author. Requests to access these datasets should be directed to graziano.onder@unicatt.it.

## Ethics statement

The studies involving human participants were reviewed and approved by Ethical Committee of the Catholic Unviersity of the Sacred Heart, approval number P/220/CE/2009. The patients/participants provided their written informed consent to participate in this study.

## Author contributions

Study concept and design: EV and RL. Acquisition of data: GO. Analysis and interpretation of data: EV, RL, and KP. Preparation of the manuscript: MZ, EV, KP, and RL. Revision of the manuscript: LS, AC, DFi, MD, DFu, and MC. All authors contributed to the article and approved the submitted version.
